# T-Type Ca^2+^ Channel Blocker, KYS05090 Induces Autophagy and Apoptosis in A549 Cells through Inhibiting Glucose Uptake

**DOI:** 10.3390/molecules19079864

**Published:** 2014-07-08

**Authors:** Hong-Kun Rim, Sehyeon Cho, Dong-Hyun Shin, Kyung-Sook Chung, Young-Wuk Cho, Jung-Hye Choi, Jae Yeol Lee, Kyung-Tae Lee

**Affiliations:** 1 Department of Pharmaceutical Biochemistry, College of Pharmacy, Kyung Hee University, 1 Hoegi-dong, Dongdaemun-gu, Seoul 130-701, Korea; E-Mails: rhk000@hanmail.net (H.-K.R.); shane5@hanmail.net (D.-H.S.); adella76@hanmail.net (K.-S.C.); 2 Department of Life and Nanopharmaceutical Science, College of Pharmacy, Kyung Hee University, 1 Hoegi-dong, Dongdaemun-gu, Seoul 130-701, Korea; E-Mail: jchoi@khu.ac.kr; 3 Department of Biomedical Science, College of Medical Science, Kyung Hee University, 1 Hoegi-dong, Dongdaemun-gu, Seoul 130-701, Korea; E-Mail: ywcho@khu.ac.kr; 4 Research Institute for Basic Sciences and Department of Chemistry, College of Sciences, Kyung Hee University, 1 Hoegi-dong, Dongdaemun-gu, Seoul 130-701, Korea; E-Mail: cm2u@naver.com; 5 Department of Molecular Biology, College of Pharmacy, Kyung Hee University, Seoul 130-701, Korea

**Keywords:** T-type Ca^2+^ channel, glucose, autophagy, apoptosis, reactive oxygen species

## Abstract

It has been reported that [3-(1,1'-biphenyl-4-yl)-2-(1-methyl-5-dimethylamino-pentylamino)-3,4-dihydroquinazolin-4-yl]-*N*-benzylacetamide 2hydrochloride (KYS05090), a selective T-type Ca^2+^ channel blocker, reduces tumor volume and weight in the A549 xenograft model, but the molecular mechanism of cell death has not yet been elucidated. In this study, KYS05090 induced autophagy- and apoptosis-mediated cell death in human lung adenocarcinoma A549 cells. Although KYS05090 decreased intracellular Ca^2+^ levels, it was not directly related with KYS05090-induced cell death. In addition, KYS05090 generated intracellular reactive oxygen species (ROS) and reduced glucose uptake, and catalase and methyl pyruvate prevented KYS05090-induced cell death. These results indicate that KYS05090 can lead to autophagy and apoptosis in A549 cells through ROS generation by inhibiting glucose uptake. Our findings suggest that KYS05090 has potential chemotherapeutic value for the treatment of lung cancer.

## 1. Introduction

Calcium is an essential signal transduction element in the progression of the cell cycle [1]. Controlling intracellular Ca^2+^ ([Ca^2+^]_i_) is crucial for the orderly progression of the cell cycle and plays a vital role in the regulation of cell proliferation and growth [2]. The T-type Ca^2+^ channels are one of the voltage-dependent Ca^2+^ channels and a low-voltage-activated Ca^2+^ channel. The T-type Ca^2+^ channel is thought to be responsible for neuronal oscillatory activity, which is proposed to be involved in processes, such as sleep/wakefulness regulation, motor coordination, and neuronal circuit specification during ontogenesis [3]. In addition, the T-type Ca^2+^ channel has been reported to be involved in pacemaker activity, pain processing, and tumor pathophysiology [4,5]. Previous studies have reported that the T-type Ca^2+^ channel is expressed in cancerous cells, and several evidences have provided new insights into the development of T-type Ca^2+^ channel blockers in cancer therapy [5,6,7].

Our previous studies have indicated that KYS05047, a T-type Ca^2+^ channel blocker, induces G_1_ phase cell cycle arrest in A549 cells associated with a decrease in [Ca^2+^]_i_ and that both KYS05047 and KYS05090 show potent *in vivo* antitumor activity against the A549 xenograft mice [8,9]. The present study examines the molecular mechanism of KYS05090 underlying cell death in A549 cells.

## 2. Results and Discussion

### 2.1. KYS05090 Induced Cell Death in A549 Cells

**Figure 1:**
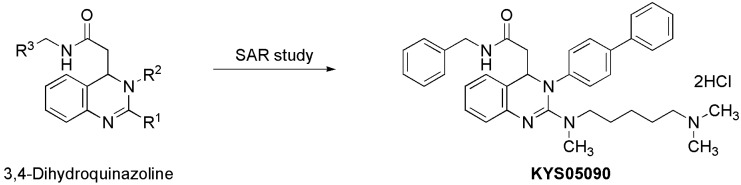
Discovery of KYS05090 via intensive structure-activity relationship (SAR) study on 3,4-dihydroquinazoline derivatives.

KYS05090 (Figure 1) was prepared as described previously [8]. To investigate the cytotoxic effect of KYS05090, viable A549 cell numbers were analyzed with the presence of different concentrations (0, 4, 6, 8, or 10 μM) of KYS05090 for 24 h by an MTT assay.

**Figure 2:**
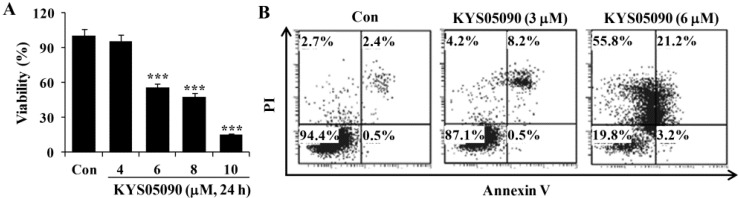
KYS05090 induced cell death in A549 cells. (**A**) A549 cells were treated with KYS05090 (0, 4, 6, 8, or 10 μM) for 24 h. Cell viability was determined through an MTT assay. Presented data are means ± S.D. for three independent experiments. *** *p* < 0.001 *vs.* the control group. (**B**) Cells were stained with Annexin V-FITC and PI and analyzed by FACS.

As shown in Figure 2A, KYS05090 (IC_50_: 6 μM) reduced cell viability in a dose-dependent manner. To identify the molecular mechanism underlying cell death induced by KYS05090, the translocation of phosphatidylserine was assessed using Annexin V and propidium iodide (PI) double staining by flow cytometry. As shown in Figure 2B, KYS05090 (6 μM for 24 h) increased both non-apoptotic and apoptotic cell death by up to 55% and 24%, respectively.

### 2.2. KYS05090 Induced Autophagy and Apoptosis in A549 Cells

**Figure 3:**
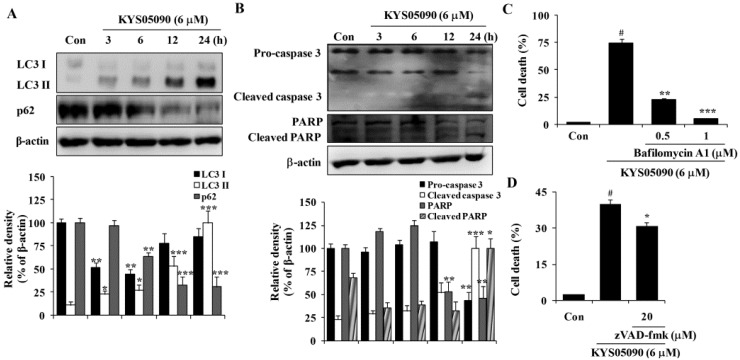
KYS05090 induced autophagy-associated and apoptotic cell death in A549 cells. (**A**,**B**) A549 cells were treated with KYS05090 (6 μM) for indicated times. The expression of proteins were analyzed by western blot analysis. β-Actin was used as an internal control. The immunoblots shown are representative of three independent experiments. Density ratios *versus* β-actin were measured by densitometry. * *p* < 0.05, ** *p* < 0.01, *** *p* < 0.001 *vs.* the control group (**C**) A549 cells were pretreated with bafilomycin A1 (0.5 or 1 μM) for 30 min and then treated with KYS05090 (6 μM) for 24 h. The cells were stained with PI, and cell death was analyzed by FACS. (**D**) A549 cells were pretreated with zVAD-fmk (20 μM) for 30 min and then treated with KYS05090 (6 μM) for 24 h. The cells were stained with PI, and cell death was analyzed by FACS. Presented data are means ± S.D. for three independent experiments. **^#^** *p* < 0.05 *vs.* the control group, and * *p* < 0.05, ** *p* < 0.01, *** *p* < 0.001 *vs.* the KYS05090-treated group.

Autophagy is thought to be a cytoprotective process in starving cells. However, excess autophagy can induce type II programmed cell death (autophagy-associated cell death) [10]. Because KYS05090 induced non-apoptotic cell death, its ability to induce autophagy-associated cell death was examined. LC3 is localized in the cytoplasm under normal conditions but is cleaved and lipidated to be turned into LC3 II and recruited to the autophagosomes during autophagy [10]. Therefore, amount and localization of LC3 II is used as marker of autophagy induction. KYS05090 induced LC3-II conversion in A549 cells in a time-dependent manner (Figure 3A). In addition, the autophagic flux was verified by the decreased expression of p62, which can be degraded by autophagy (Figure 3A). To verify the involvement of autophagy in KYS05090-induced cell death, bafilomycin A1 was used at concentrations that significantly blocked the induction of autophagy. As shown in Figure 3C, bafilomycin A1 markedly suppressed KYS05090-induced cell death. These observations indicate that KYS05090-induced cell death involves the autophagy-dependent pathway in A549 cells.

Annexin V/PI double-staining results indicate that KYS05090 mildly induced apoptosis. To characterize KYS05090-triggered apoptosis, the activation of caspase 3 in A549 cells by KYS05090 treatment was examined by western blotting. Pro-caspase 3 was activated after KYS05090 treatment, and this induction was accompanied by an increase in the cleavage of its substrate poly (ADP-ribose) polymerase (PARP) (Figure 3B). To determine whether the activation of caspases is required for the induction of apoptosis by KYS05090, the broad caspase inhibitor (zVAD-fmk) was pretreated in A549 cells. As shown in Figure 3D, zVAD-fmk significantly inhibited KYS05090-induced cell death. These results indicate that KYS05090 induced caspase-dependent apoptotic cell death as well as autophagy-associated cell death.

### 2.3. KYS05090 Reduced [Ca^2+^]_i_ but Was Not Related to KYS05090-Induced Cell Death in A549 Cells

**Figure 4:**
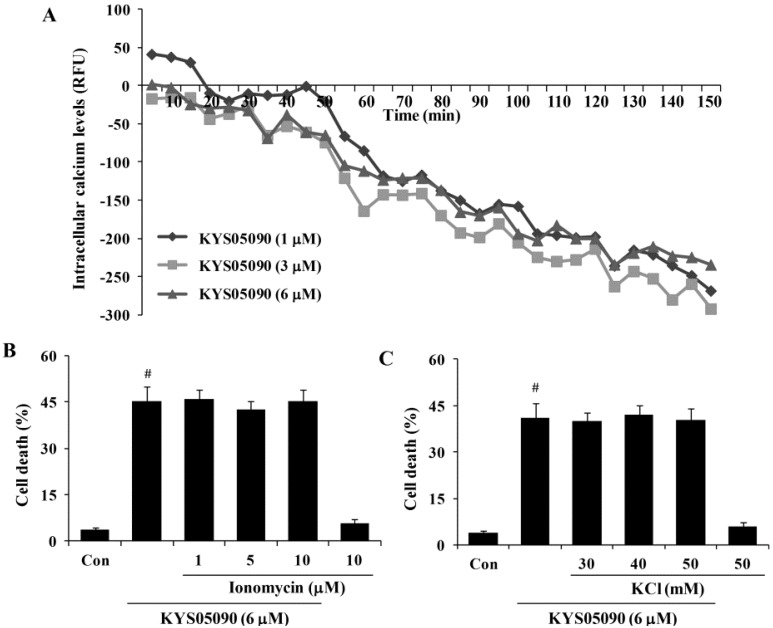
KYS05090 reduced [Ca^2+^]_i_ but was not associated with KYS05090-induced cell death in A549 cells. (**A**) A549 cells were stained with Fluo-4 and treated with KYS05090 (1, 3, 6, 8, or 10 μM). [Ca^2+^]_i_ were measured using a fluorescence reader. (**B**,**C**) A549 cells were pretreated with ionomycin (1, 5, or 10 μM) or KCl (30, 40, or 50 mM) for 30 min and then treated with KYS05090 (6 μM) for 24 h. The cells were stained with PI, and cell death was analyzed by FACS. Presented data are means ± S.D. for three independent experiments. **^#^** *p* < 0.05 *vs.* the control group.

To identify the relationship between cell death induced by KYS05090 and its ability to block the Ca^2+^ channel, the dependence of KYS05090-induced cell death on the [Ca^2+^]_i_ was examined. KYS05090 treatment (1, 3, or 6 μM) reduced [Ca^2+^]_i_ in a time-dependent manner. To prevent KYS05090-induced cell death by a decrease in [Ca^2+^]_i_, the effect of ionomycin or KCl, which increases [Ca^2+^]_i_, was examined [11], and the results indicate that these [Ca^2+^]_i_ enhancers did not prevent KYS05090-induced cell death in A549 cells (Figure 4B,C). These data indicated that KYS05090-induced decrease in [Ca^2+^]_i_ were not related to KYS05090-induced cell death.

### 2.4. Reactive Oxygen Species (ROS) Were Involved in KYS05090-Induced Cell Death

**Figure 5:**
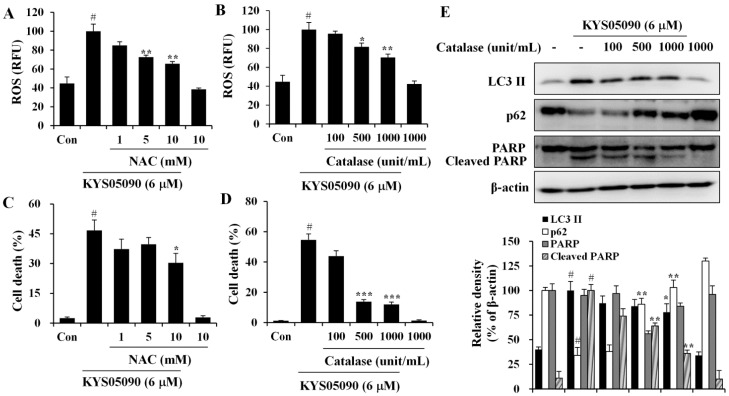
KYS05090 generated ROS, and antioxidants inhibited KYS05090-induced cell death. (**A**,**B**) A549 cells were pretreated with NAC or catalase for 30 min, followed by treatment with KYS05090 (6 μM) for 30 min and DCFH-DA (5 µM) for 30 min. Subsequently, the cells were collected and analyzed by FACS. (**C**,**D**) A549 cells were pretreated with NAC or catalase for 30 min and then treated with KYS05090 (6 μM) for 24 h. The cells were stained with PI, and cell death was analyzed by FACS. Presented data are means ± S.D. for three independent experiments. **^#^** *p* < 0.05 *vs.* the control group, and * *p* < 0.05, ** *p* < 0.01, *** *p* < 0.001 *vs.* the KYS05090-treated group. (**E**) A549 cells were pretreated with catalase for 30 min and then treated with KYS05090 (6 μM) for 24 h. Protein levels were analyzed by western blot analysis. β-Actin was used as an internal control. The immunoblots shown are representative of three independent experiments. Density ratios *versus* β-actin were measured by densitometry. **^#^** *p* < 0.05 *vs.* the control group, and * *p* < 0.05, ** *p* < 0.01 *vs.* the KYS05090-treated group.

Because ROS has been demonstrated to mediate autophagy as well as apoptosis [12], its involvement in KYS05090-mediated cell death was examined. The level of ROS within cells was measured using a ROS-sensitive fluoromertric probe, DCFH-DA, and flow cytometry. As shown in Figure 5A,B, cells treated with KYS05090 showed higher levels of ROS production in comparison to control cells, and the KYS05090-induced ROS generation was dose-dependently reduced by the antioxidant NAC or catalase. In addition, treatment with these antioxidants effectively blocked KYS05090-induced cell death, and noteworthy is that catalase was significantly more likely to prevent this cell death than NAC (Figure 5C,D). Furthermore, catalase prevented KYS05090-induced LC3-II accumulation, p62 degradation, and PARP cleavage (Figure 5E). These results suggest that ROS play a critical role in KYS05090-induced cell death.

### 2.5. KYS05090 Induced Autophagy and Apoptosis by Suppressing Glucose Uptake in A549 Cells

**Figure 6:**
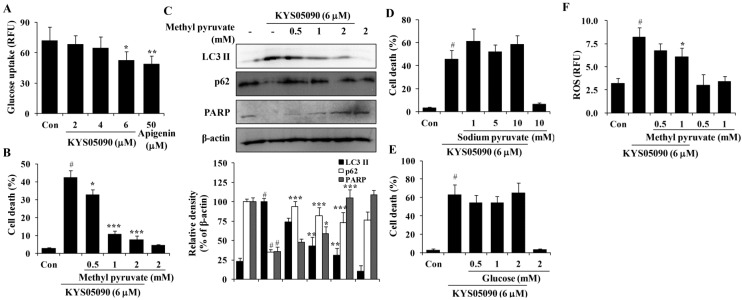
KYS05090 suppressed glucose uptake, and methyl pyruvate inhibited KYS05090-induced autophagy-associated and apoptotic cell death in A549 cells. (**A**) A549 cells (5 × 10^4^/mL) were seeded in a 96-well plate and treated with KYS05090 (2, 4, or 6 μM) in the glucose- and serum-free culture medium. After 2 h, 100 µg/mL of 2-NBDG was added, and the plates were incubated for an additional 2 h. Fluorescence intensity was measured using a fluorescence plate reader. Apigenin was used as a positive control. Presented data are means ± S.D. for three independent experiments. * *p* < 0.05 and ** *p* < 0.01 *vs.* the control group. (**B**–**E**) A549 cells were pretreated with methyl pyruvate (0.5, 1, or 2 mM), sodium pyruvate (1, 5, or 10 mM), or glucose (0.5, 1, or 2 mM) for 30 min and then treated with KYS05090 (6 μM) for 24 h. The cells were stained with PI, and cell death was analyzed by FACS. Protein levels were analyzed by western blot analysis. β-Actin was used as an internal control. The immunoblots shown are representative of three independent experiments. Density ratios *versus* β-actin were measured by densitometry. Presented data are means ± S.D. for three independent experiments. **^#^** *p* < 0.05 *vs.* the control group, and * *p* < 0.05, ** *p* < 0.01, *** *p* < 0.001 *vs.* the KYS05090-treated group. (**F**) A549 cells were pretreated with methyl pyruvate (0.5, 1, or 2 mM) for 30 min, followed by treatment with KYS05090 (6 μM) for 30 min and DCFH-DA (5 µM) for 30 min. Then the cells were collected and analyzed by FACS. Presented data are means ± S.D. for three independent experiments. **^#^** *p* < 0.05 *vs.* the control group, and * *p* < 0.05 *vs.* the KYS05090-treated group.

Several studies have reported that Ca^2+^ channel antagonists inhibit glucose uptake and that glucose deprivation subsequently induces oxidative stress associated with aggresome formation and autophagy activation [13,14,15]. In addition, ROS inhibition by catalase can prevent cell death induced by glucose deprivation [16]. Therefore, the ability of KYS05090 to inhibit glucose uptake, as in other Ca^2+^ channel antagonists, was examined, and the results indicate that KYS05090 dose-dependently inhibited glucose uptake in A549 cells (Figure 6A). To determine whether KYS05090 would induce cell death through the inhibition of glucose uptake, A549 cells were pretreated with methyl pyruvate (cell permeable pyruvate). As shown in Figure 6B, methyl pyruvate significantly inhibited KYS05090-induced cell death. In addition, methyl pyruvate prevented KYS05090-induced LC3-II accumulation, p62 degradation, and PARP cleavage (Figure 6C). By contrast, sodium pyruvate (cell impermeable pyruvate) or glucose did not attenuate KYS05090-induced cell death (Figure 6D,E). These results indicate that KYS05090-induced cell death was caused by the downregulation of glucose uptake.

It has been reported that glucose deprivation can generate ROS [15]. To investigate the relationship between KYS05090-induced ROS generation and the inhibition of glucose uptake, KYS05090-induced ROS generation was determined after the treatment of A549 cells with methyl pyruvate. As shown in Figure 6F, methyl pyruvate (1 mM) significantly reduced KYS05090-induced ROS generation. This indicates that KYS05090 generated ROS through the inhibition of glucose uptake. Methyl pyruvate (1 mM) prevented KYS05090-induced cell death more powerfully than 0.5 mM of methyl pyruvate (Figure 6B). However, the difference in levels of p62 or ROS are less than cell death level (Figure 6C,F). These results give a possibility that KYS05090-induced cell death may be partially related with autophagy and ROS generation.

## 3. Experimental

### 3.1. Materials

RPMI 1640 medium, fetal bovine serum (FBS), penicillin, and streptomycin were obtained from Invitrogen (Carlsbad, CA, USA). Antibodies for caspase 3, PARP, p62, and β-actin were purchased from Santa Cruz Biotechnology (Santa Cruz, CA, USA). Antibody for LC3 I/II was purchased from Cell Signaling Technology (Danvers, MA, USA). zVAD-fmk and ionomycin were purchased from Calbiochem (Bad Soden, Germany). MTT, dimethyl sulfoxide (DMSO), methyl pyruvate, sodium pyruvate, glucose, KCl, catalase, bafilomycin A1, NAC, RNase A, leupeptin, aprotinin, phenylmethylsulfonylfluoride (PMSF), Triton X-100, and PI and all other chemicals were purchased from Sigma (St. Louis, MO, USA).

### 3.2. Cell Culture

Human lung adenocarcinoma A549 cells were obtained from the Korean cell line bank (Seoul, Korea). Cells were cultured in RPMI 1640 supplemented with 10% heat-inactivated FBS, penicillin (100 U/mL) and streptomycin sulfate (100 µg/mL). Cells were cultured at 37 °C in an atmosphere of 5% CO_2_.

### 3.3. MTT Assay

The cells (5 × 10^4^/mL) were seeded in each well containing 100 µL of the RPMI medium supplemented with 10% FBS in a 96-well plate. Various concentrations of KYS05090 were added and incubated for 24 h. MTT (5 mg/mL stock solution) was added and the plates were incubated for an additional 4 h. The medium was discarded and the formazan blue, which was formed in the cells, was dissolved with 100 µL DMSO. The optical density was measured at 540 nm by an automatic microplate reader (Molecular Devices Corp., Sunnyvale, CA, USA) [17].

### 3.4. Annexin V and PI Double Staining by Flow Cytometry

Cells were treated with various concentrations of KYS05090, washed with 1 mL phosphate buffered saline (PBS), suspended with 100 mL of binding buffer (10 mM HEPES/NaOH, 140 mM NaCl, 2.5 mM CaCl_2_, pH 7.4), and stained with 5 µL of FITC-conjugated Annexin V and 5 µL of PI (50 mg/mL). The mixture was incubated for 15 min at room temperature in dark place and analyzed by the flow cytometry [18].

### 3.5. Cell Death Analysis

Cells were washed with 1 mL PBS, fixed in 70% ice-cold ethanol and kept in a freezer overnight. The fixed cells were centrifuged, washed twice with PBS and re-suspended in PBS containing 50 mg/mL PI and 100 μg/mL DNase-free RNase A. The cell suspension, which was hidden from light, was incubated for 30 min and analyzed using the flow cytometry.

### 3.6. Western Blot Analysis

Cells were collected by centrifugation and washed once with PBS. The washed cell pellets were resuspended in extraction lysis buffer (50 mM HEPES pH 7.0, 250 mM NaCl, 5 mM EDTA, 0.1% Nonidet P-40, 1 mM PMSF, 0.5 mM DTT, 5 mM Na fluoride, and 0.5 mM Na orthovanadate) containing 5 µg/mL each of leupeptin and aprotinin and incubated with 20 min at 4 °C. Cell debris was removed by microcentrifugation, followed by quick freezing of the supernatants. The protein concentration was determined using the Bio-Rad protein assay reagent, according to the manufacturer’s instructions. Cellular protein was electroblotted onto a PVDF membrane following separation on a SDS-polyacrylamide gel electrophoresis. The immunoblot was incubated with blocking solution (5% skim milk) for 1 h, followed by incubation overnight with a primary antibody. Blots were washed three times with Tween 20/Tris-buffered saline (T/TBS) and incubated with a 1:3000 dilution of horseradish peroxidase-conjugated secondary antibody for 2 h at room temperature. Blots were again washed three times with T/TBS, and then developed by enhanced chemiluminescence (Amersham Life Science, Arlington Heights, IL, USA) [19].

### 3.7. Measurement of [Ca^2+^]_i_

[Ca^2+^]_i_ was measured using Flou-4 calcium assay kit (Invitrogen), according to the manufacturer’s instructions. A549 cells (5 × 10^4^/mL) were seeded in each well containing 100 µL of the RPMI medium supplemented with 10% FBS in a 96-well plate. The next day, medium were removed and add 100 μL of the Flou-4 containing assay solution to each well. Incubate the plate at 37 °C for 30 min and then cells were treated with vehicle or various concentrations of KYS05090 and fluorescence intensities were measured using a fluorescence plate reader (Hitachi, Tokyo, Japan).

### 3.8. Glucose Uptake Assay

The glucose uptake was measured using 2-deoxy-2-[(7-nitro-2,1,3-benzoxadiazol-4-yl) amino]-d-glucose (2-NBDG, Cayman Chemical Company, Ann Arbor, MI, USA), according to the manufacturer’s instructions. Briefly, A549 cells (5 × 10^4^/mL) were seeded in each well containing 100 µL of the RPMI medium supplemented with 10% FBS in a 96-well plate. The next day, cells were treated with vehicle or various concentrations of KYS05090 in glucose- and serum-free culture medium. After 2 h, 100 µg/mL of 2-NBDG was added and the plates were incubated for an additional 2 h. The fluorescence intensities were measured using a fluorescence plate reader. Apigenin was used as a positive control [20].

### 3.9. Determination of ROS Generation

Generation of intracellular ROS was examined by flow cytometry using 2',7'-dichlorofluorescin diacetate (DCFH-DA; Molecular Probes). Briefly, A549 cells (5 × 10^4^/mL) were seeded plates and allowed to attach overnight. The cells were first exposed to KYS05090 for 30 min and then treated with 5 µM DCFH-DA for 30 min at 37 °C. Subsequently, the cells were collected by trypsinization, washed twice with PBS, and analyzed for dichlorodihydrofluorescein fluorescence using flow cytometry [21].

### 3.10. Statistical Analysis

All data are presented as the mean ± standard deviation (SD). Experiments were performed three times independently. Statistical significances were determined using ANOVA and Dunnett’s post-hoc test. Statistical significance was set at *p* < 0.05.

## 4. Conclusions

KYS05090, a T-type Ca^2+^ channel blocker, had a cytotoxic effect through the induction of autophagy and apoptosis through ROS generation based on the inhibition of glucose uptake. These results suggest that T-type Ca^2+^ channel blockers may be drug candidates for anticancer therapy.
